# Supplementary material for: Dorsal hippocampus to nucleus accumbens projections drive reinforcement via activation of accumbal dynorphin neurons
Source: Nat Commun. 2024 Jan 29;15:750. doi: 10.1038/s41467-024-44836-9 (PMC10825206; doi:10.1038/s41467-024-44836-9)
Supplement: Supplementary file 1 — Supplementary Information [file 41467_2024_44836_MOESM1_ESM.pdf]

Dorsal Hippocampus To Nucleus Accumbens Shell Projections Drive Reinforcement Via Activation of Accumbal Dynorphin Neurons

Khairunisa Mohamad Ibrahim [1,2,*], Nicolas Massaly [1,2,*], Hye-Jean Yoon [1,2], Rossana Sandoval [1,2,3], Allie Widman [1,2], Robert J. Heuermann [1,2], Sidney Williams [1,2], William Post [1,2], Sulan Pathiranage [1,2], Tania Lintz [1,2], Azra Zec [1,2], Ashley Park [1,2], Waylin Yu [4,5], Thomas L. Kash [4,5], Robert W. Gereau IV [1,2,7], Jose A. Morón [1,2,6,7,#]

[1] Department of Anesthesiology, Washington University Pain Center, St. Louis, MO 63110, USA
[2] Washington University in St. Louis, School of Medicine, St. Louis, MO 63110, USA
[3] St. Louis University, St. Louis, MO 63103, USA
[4] Bowles Center for Alcohol Studies, University of North Carolina at Chapel Hill School of Medicine, Chapel Hill, NC 27599, USA
[5] Department of Pharmacology, University of North Carolina at Chapel Hill School of Medicine, Chapel Hill, NC 27599, USA
[6] Department of Psychiatry, Washington University in St. Louis, St. Louis, MO 63110, USA
[7] Department of Neuroscience, Washington University in St. Louis, St. Louis, MO 63110, USA

*These authors contributed equally to this work

Lead Contact: Jose A. Morón : jmoron-concepcion@wustl.edu

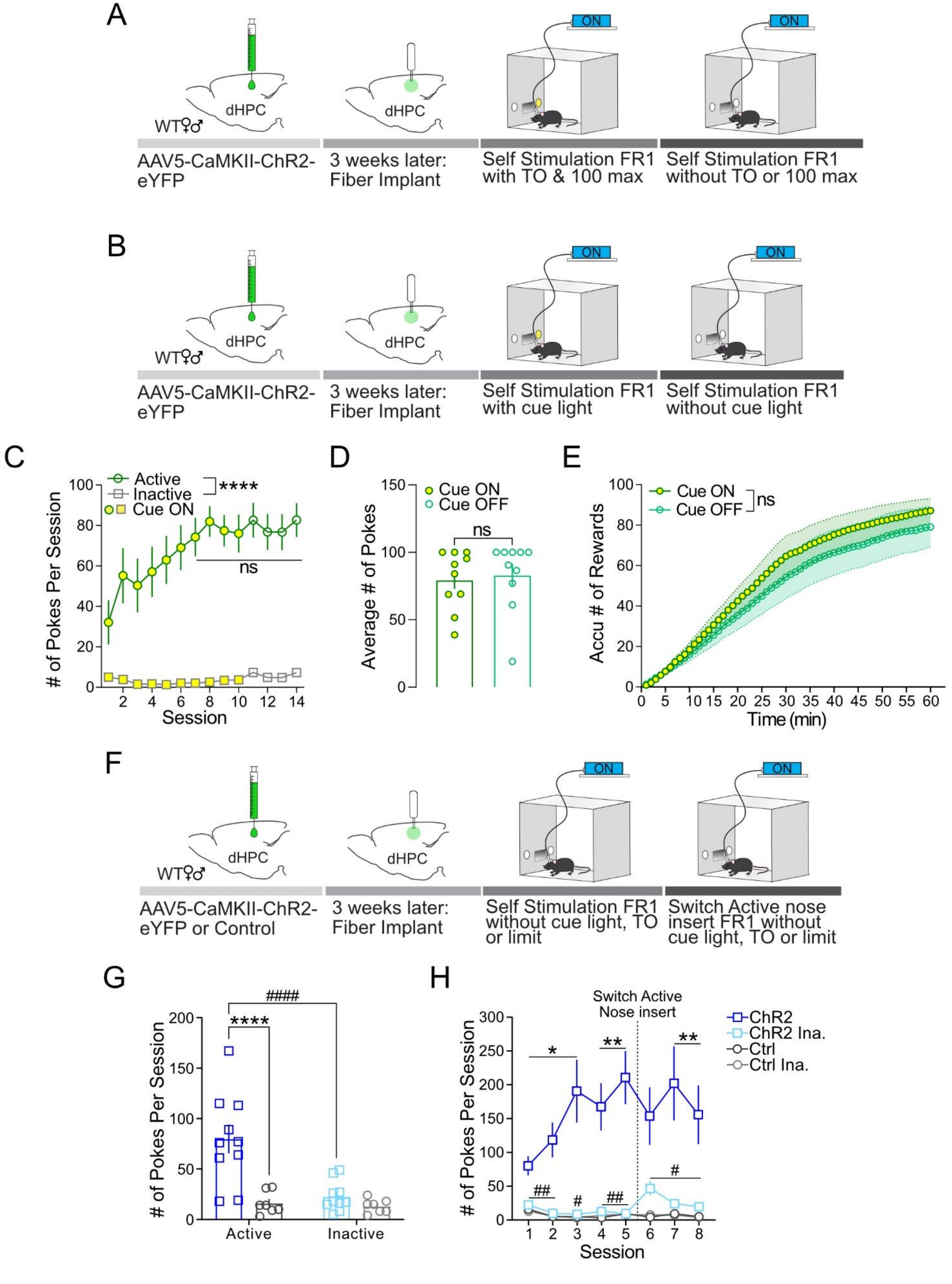

A
ON
dHPC
WT♀♂
AAV5-CaMKII-ChR2-eYFP
3 weeks later: Fiber Implant
Self Stimulation FR1 with TO & 100 max
Self Stimulation FR1 without TO or 100 max
B
AAV5-CaMKII-ChR2-eYFP
3 weeks later: Fiber Implant
Self Stimulation FR1 with cue light
Self Stimulation FR1 without cue light
C
Active
Inactive
Cue ON
****
ns
# of Pokes Per Session
Session
D
Cue ON
Cue OFF
ns
Average # of Pokes
E
Cue ON
Cue OFF
ns
Accu # of Rewards
Time (min)
F
AAV5-CaMKII-ChR2-eYFP or Control
3 weeks later: Fiber Implant
Self Stimulation FR1 without cue light, TO or limit
Switch Active nose insert FR1 without cue light, TO or limit
G
####
****
# of Pokes Per Session
Active
Inactive
H
Switch Active Nose insert
ChR2
ChR2 Ina.
Ctrl
Ctrl Ina.
# of Pokes Per Session
Session

Supplementary figure 1: **Stimulation of dHPC CaMKII^{+} neurons is not cue dependent.**

(A) Experimental schematic outlining dHPC self-stimulation in the absence of time out (TO) and max limit of 100 stimulations/session (max 100). **(B)** Experimental schematic outlining dHPC self-stimulation in the absence of cue. **(C)** Mice continue to discriminate between the active (green) and inactive (grey) insets in the presence of cue light (n=10; Active vs Inactive: Two-way ANOVA, $F_{1,11} = 79.25$, $p < 0.0001$ with Tukey's multiple comparison). Mice maintained the number of active pokes in the absence of cue light (last 4 sessions). **(D)** The average number of active pokes in the last 4 sessions with cue light was comparable with the average number of active pokes in the 4 sessions without the cue light (Cue vs No cue: two-tailed Wilcoxon matched-pairs signed rank test, $p = 0.7422$). **(E)** The accumulation number of rewards obtained within the last session with cue light is no different from the last session without the cue light (Cue vs No cue: Two-way ANOVA, $F_{1,9} = 4.539$, $p = 0.0620$ with Sidak's multiple comparison). **(F)** Experimental schematic outlining dHPC stimulation in the absence of cue light, time out (TO) and max limit. Nose inset that is paired with dHPC stimulation is labelled as active while the other is inactive. **(G)** ChR2-expressing mice showed significantly higher number of active pokes vs inactive pokes on day 1 (n=10; Active vs Inactive: Two-way ANOVA, $F_{1,15} = 14.68$, $p = 0.0016$ with uncorrected Fisher's LSD). These mice also showed a significantly higher active pokes compared to control mice (ChR2 vs control: Two-way ANOVA, $F_{1,15} = 12.27$, $p = 0.0032$ with uncorrected Fisher's LSD). **(H)** The number of active pokes obtained by ChR2 mice was maintained (session 3-5). During sessions 6-8, the inset previously paired with light stimulation was switched to the one formerly inactive during sessions 1-5. ChR2-expressing mice still discriminated and obtained significantly higher dHPC stimulations compared to control mice (Two-way ANOVA, ChR2 vs Ctrl: $F_{3,30} = 20.41$, $p < 0.0001$ with Tukey's multiple comparisons; * Active vs Inactive ChR2 group and # ChR2 vs Ctrl Active). Data are expressed as mean $\pm$ S.E.M.

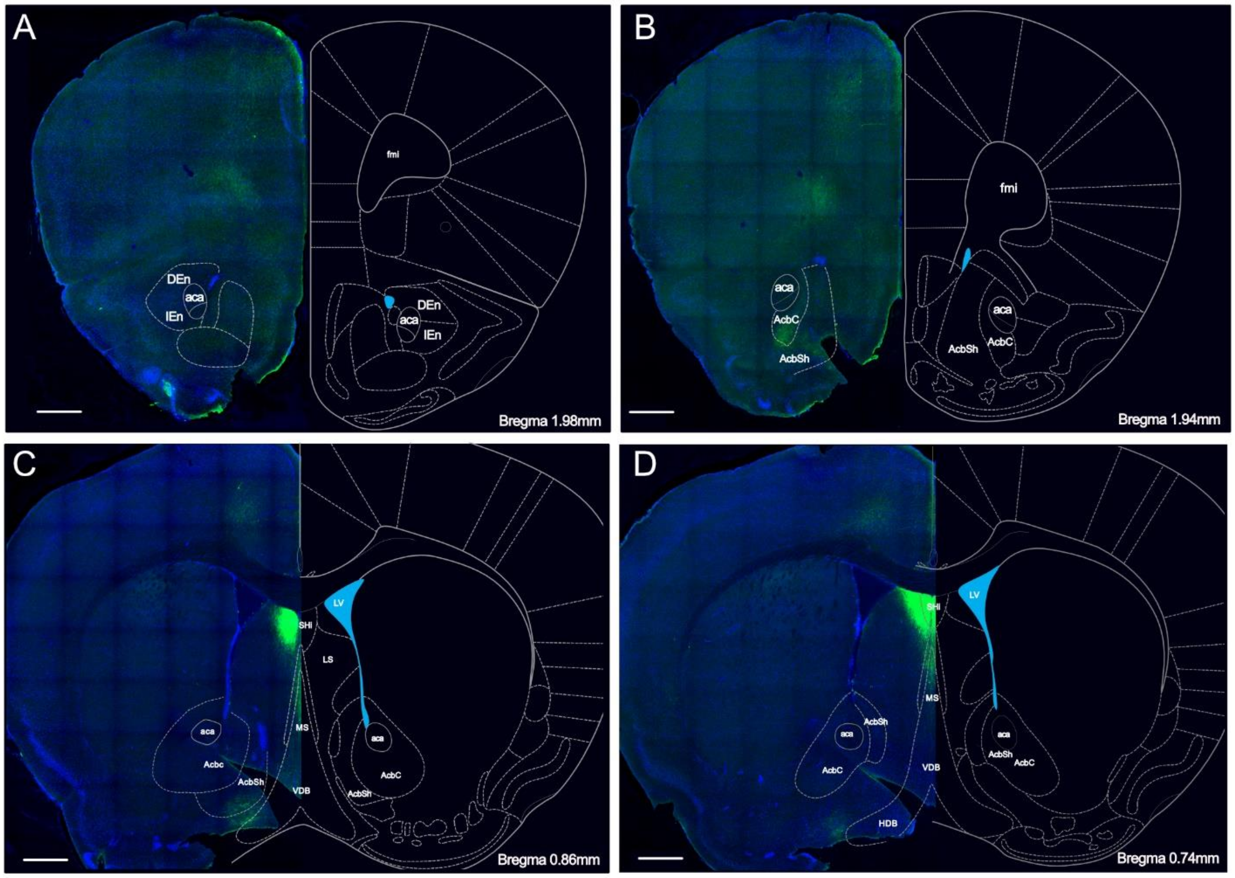

Supplementary figure 2: **dHPC CaMKII$^+$ fibers in the anterior and posterior regions of the NAc.**

(A-B) Brain sections that correspond to the anterior regions of the NAc (1.94mm to 1.98mm from bregma). **(C-D)** Brain sections that correspond to the posterior regions of the NAc (0.74mm to 0.86mm from bregma). Scale bars represent 500 μm.

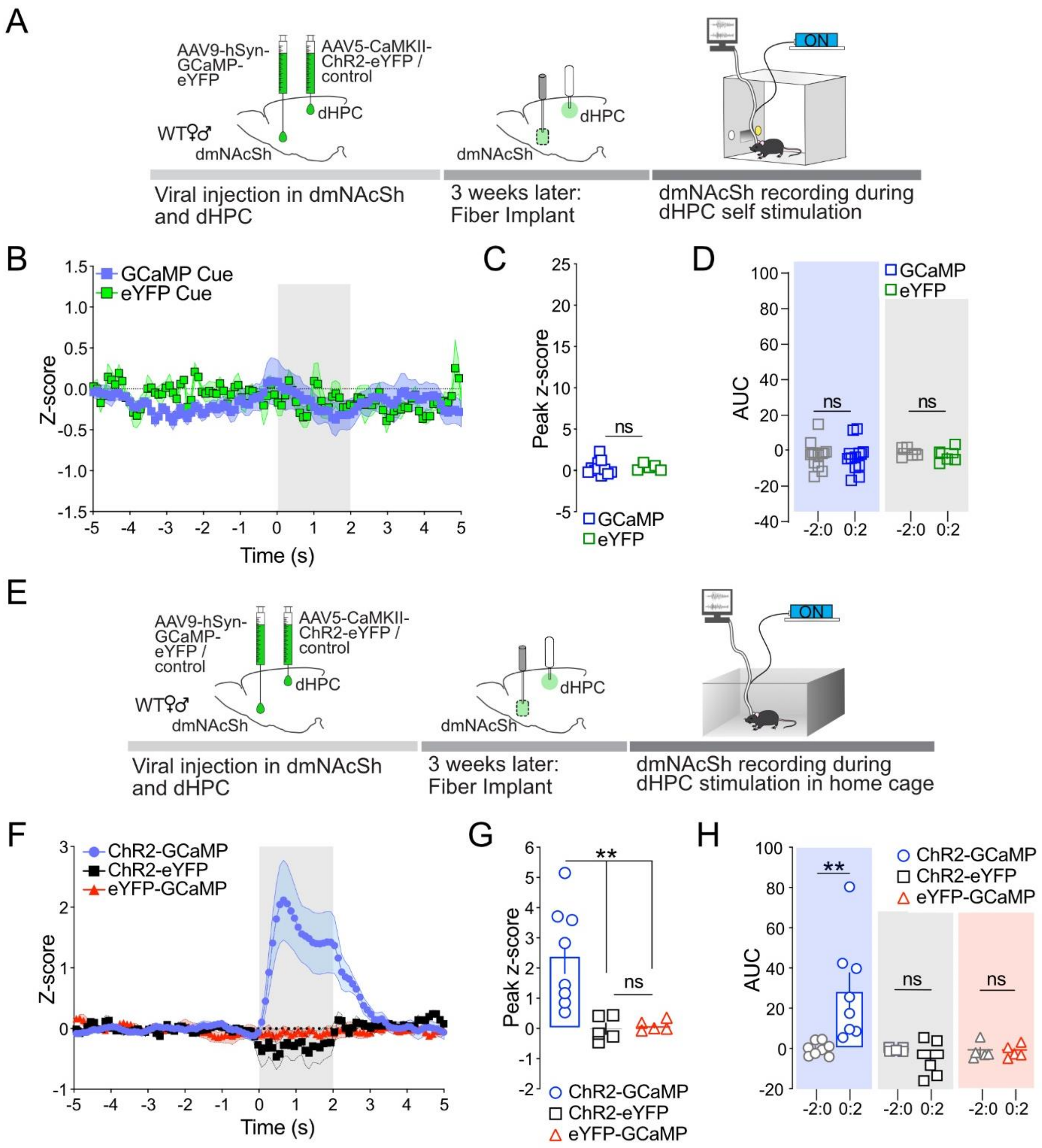

Supplementary figure 3: **Increase in dmNAcSh calcium transients is dependent on dHPC activation, but not cue associated with light stimulation.**

(**A**) Experimental schematic outlining fiber photometry recording in the dmNAcSh with dHPC stimulation in the self-stimulation operant boxes. **(B)** Time course for the calcium transient Z-scores. Cue light was on at time 0 second and the gray area represents the 2 seconds after cue light onset. The onset of cue light in the active nose inset had no effect on the

calcium transients (n=5 eYFP; 10 GCaMP). **(C)** The peak Z-score during the 2 seconds after cue light onset remains unchanged in both GCaMP- and GFP-expressing mice (GCaMP vs eYFP: two-tailed Mann Whitney, p = 0.4409). **(D)** Area under the curve (AUC) 2s after cue light onset (0:2) in GCaMP and GFP groups is similar to their respective baseline (2s before cue light onset, -2:0) (GCaMP -2:0 vs 0:2 : $t_{10} =$ 0.3159, p = 0.7586; eYFP -2:0 vs 0:2 : two-tailed Wilcoxon matched-pairs signed rank test, p = 0.8125). **(E)** Experimental schematic outlining fiber photometry recording in the dmNAcSh with experimenter-induced dHPC stimulation. **(F)** Time course for the Z-scores of the calcium transient. Calcium transients selectively increase in mice ChR2-GCaMP mice upon dHPC $CaMKII^+$ stimulations (n=5 for ChR2-eYFP; eYFP-GCaMP and 8ChR2-GCaMP). **(G)** The peak Z-score during the 2s of dHPC $CaMKII^+$ stimulation is significantly higher in ChR2-GCaMP expressing mice compared to groups that received control virus in either the dHPC (eYFP-GCaMP) or the dmNAcSh (ChR2-eYFP) (Kruskal-Wallis test followed by Dunn's multiple comparison: ChR2-GCaMP vs ChR2-eYFP p = 0.0054; ChR2-GCaMP vs eYFP-GCaMP p = 0.0157; eYFP-GCaMP vs ChR2-eYFP p > 0.9999). **(H)** Area under the curve (AUC) of 2s prior and during dHPC stimulation. The AUC of the calcium transients measured in the dmNAcSh of ChR2-GCaMP animals were significantly higher during stimulation (ChR2-GCaMP: 0:2) compared to their respective baseline (ChR2-GCaMP: -2:0) ($t_7 =$ 3.645, p = 0.0082). The AUC of the calcium transients recorded did not significantly change upon dHPC stimulation in the control groups (ChR2-GFP: two-tailed Wilcoxon matched-pairs signed rank test, p = 0.3125 and GFP-GCaMP: two-tailed Wilcoxon matched-pairs signed rank test, p > 0.9999). Data are expressed as mean ± S.E.M.

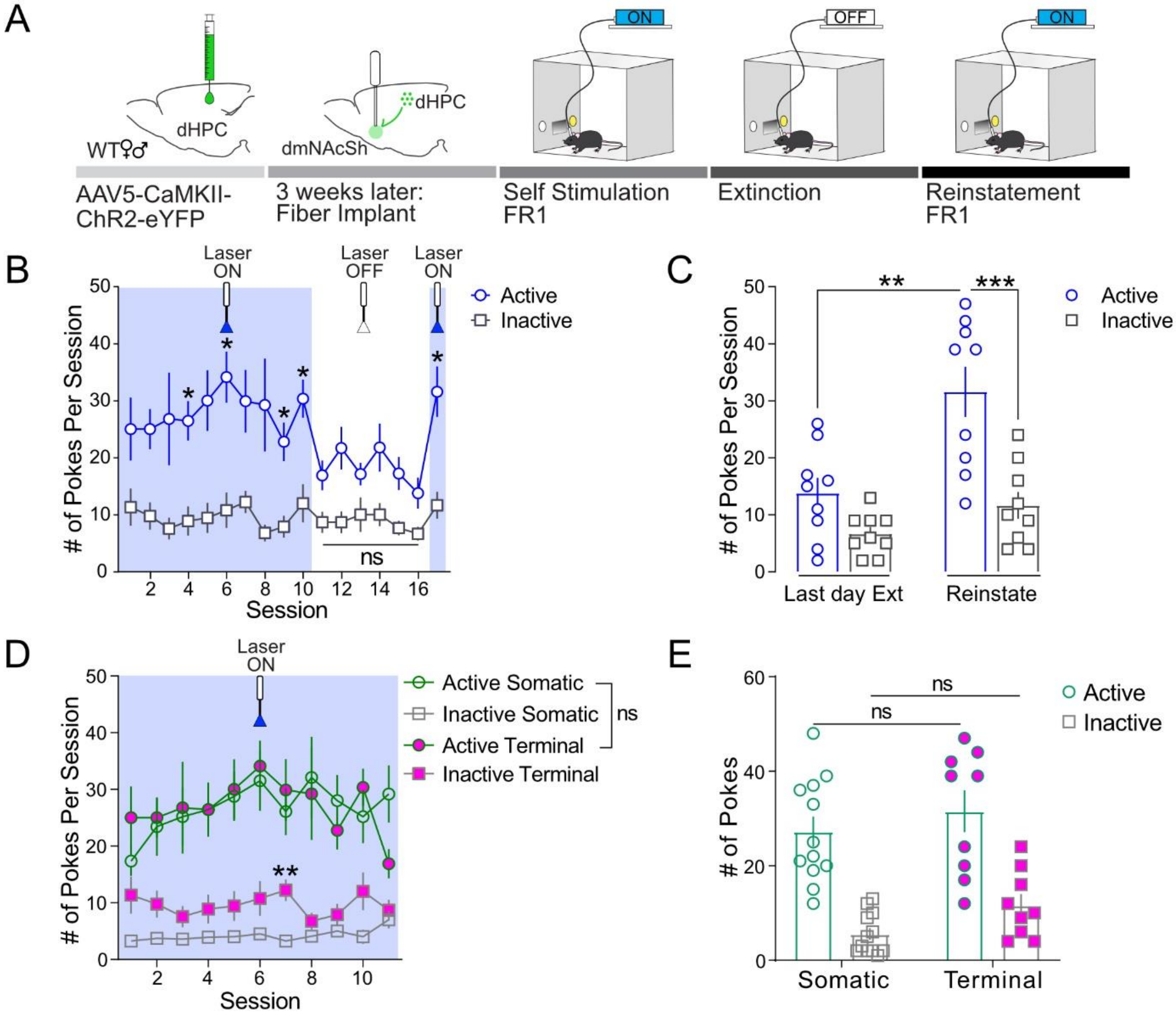

Supplementary figure 4: **dHPC-dmNAcSh terminal stimulation drives reinforcement and modulates self-stimulation.**

(A) Experimental schematic illustrating dHPC-dmNAcSh projection terminal stimulation. Briefly, CaMKII-ChR2 virus was injected in the dHPC while optic fibers were implanted in the dmNAcSh. Mice were allowed to recover before undergoing the self-stimulation protocol. **(B)** Mice expressing ChR2 within the dHPC and receiving stimulation in the dmNAcSh rapidly discriminated between the active and inactive insets, seeking stimulation through persistent active nose pokes. The persistence of reinforcing behavior stopped when the active nose poke was not associated with stimulation in the dmNAcSh (extinction sessions; laser turned OFF) (n=9). 24 hours after the last extinction session, animals were

exposed to a reinstatement session during which the laser was turned back ON. Mice interacted significantly more with the active than the inactive inset (Active vs Inactive: Two-way ANOVA, $F_{1,16} = 40.33$, $p < 0.0001$ with Sidak's multiple comparison). **(C)** Mice significantly increase their active nose pokes during reinstatement compared to the inactive nose pokes and active nose pokes during the last day of extinction (Time x Somatic vs Terminal: Two-way ANOVA, $F_{30,378} = 1.630$, $p = 0.0214$ with Sidak's multiple comparison test indicate no significance between Active Somatic and Active terminal across sessions). **(D)** Mice that received stimulation at the dHPC terminals in the dmNAcSh (Active terminal, n=9) showed similar number of active nose pokes as mice that received stimulation in dHPC that selectively projects to the dmNAcSh (Active Somatic, n=12, Figure 2**A-C**). **(E)** The number of nose pokes during reinstatement for the mice receiving stimulations in the terminal (Terminal) was no different from the mice that received stimulation in the dHPC that selectively projects to the dmNAcSh (Somatic) (Somatic vs Terminal x Active vs Inactive: Two-way ANOVA, $F_{1,19} = 0.1317$, $p = 0.7207$ with Sidak's multiple comparison). Data are expressed as mean ± S.E.M.

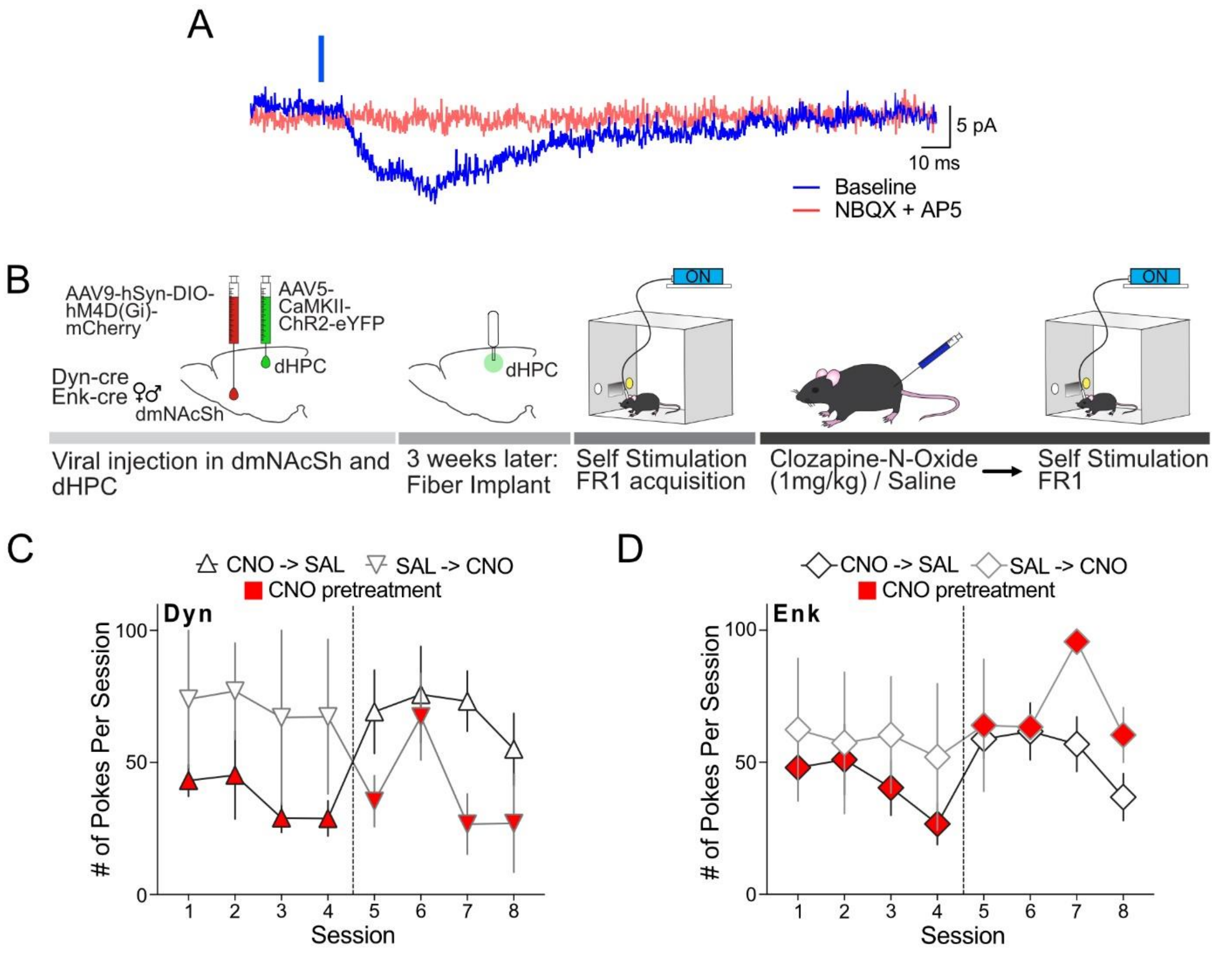

Supplementary figure 5: **Silencing dynorphin, but not enkephalin, containing neurons decreases active light self-stimulation.**

(**A**) Representative trace of the Dyn$^+$ cells with 470 nm stimulation (indicated by the blue line above the traces). The loss of 470 nm evoked responses when NBQX and AP5 was bath applied suggested that its activation was via glutamatergic input from the dHPC. **(B)** Experimental schematic outlining fiber photometry recording in the dmNAcSh dynorphin (Dyn) or enkephalin (Enk) containing neurons with non-contingent (experimenter-induced) dHPC stimulation in home cage. **(C)** Daily representation of nose pokes in the active inset per session for Dyn-cre animals. While one group of animals received 4 days of saline i.p. pretreatment before being exposed to 4 sessions with CNO i.p. pretreatment (grey downwards triangles), the other group was first exposed to four days of CNO before receiving saline i.p. pretreatment (black upwards triangles). Regardless of the days animals received CNO pre-treatment, silencing Dyn containing neurons reduces the number of pokes in the active inset (self-stimulation). **(D)** Daily representation of nose pokes in the active inset per session for Enk-cre

animals. Animals were exposed to a similar saline/CNO pretreatment schedule as mentioned above. However, silencing NAc Enk containing does not impact nose pokes in the active inset (self-stimulation). Data are expressed as mean ± S.E.M.

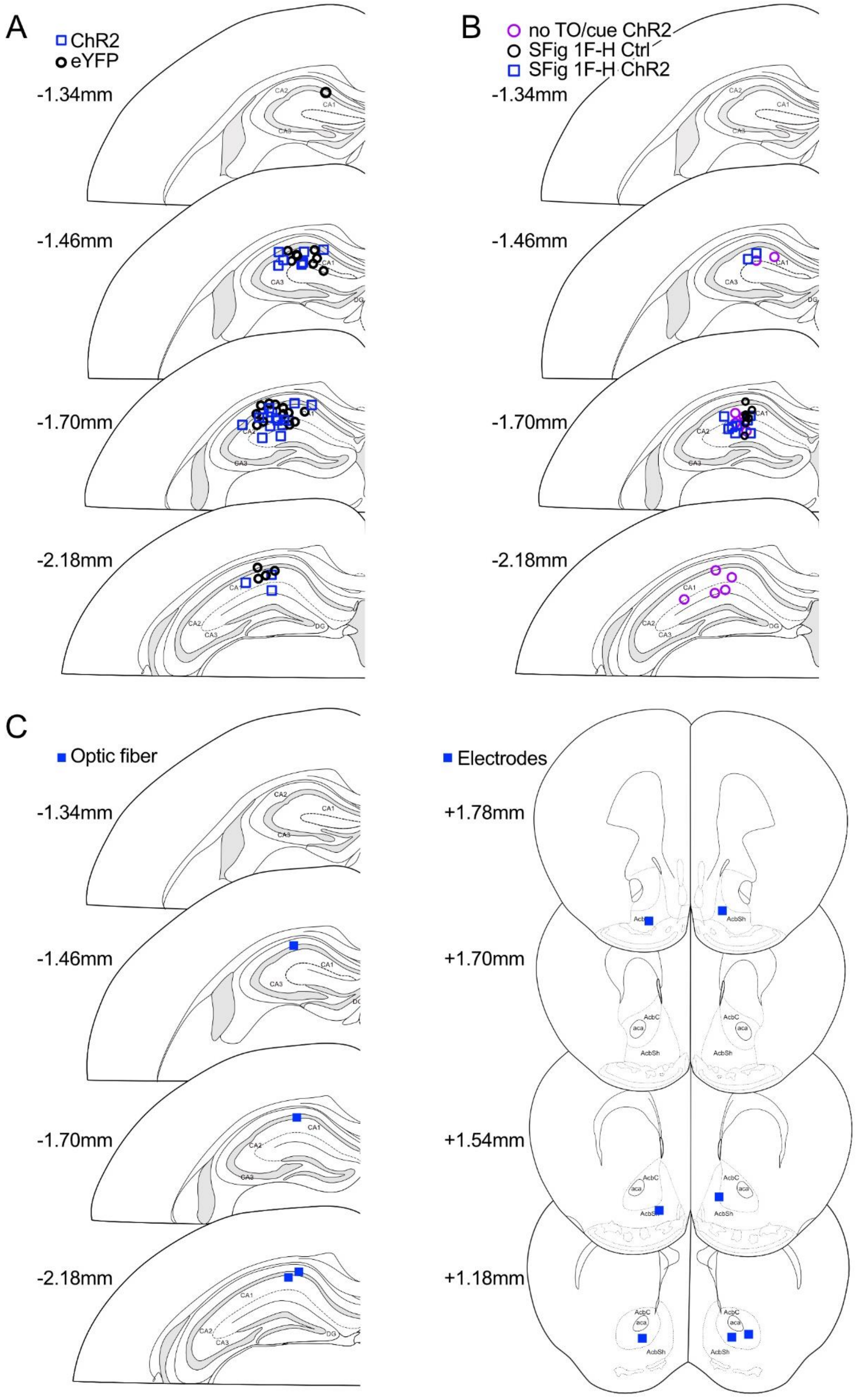
A
ChR2
eYFP
-1.34mm
-1.46mm
-1.70mm
-2.18mm
B
no TO/cue ChR2
SFig 1F-H Ctrl
SFig 1F-H ChR2
-1.34mm
-1.46mm
-1.70mm
-2.18mm
C
Optic fiber
-1.34mm
-1.46mm
-1.70mm
-2.18mm
Electrodes
+1.78mm
+1.70mm
+1.54mm
+1.18mm
CA1
CA2
CA3
DG
AcbC
AcbSh
aca

Supplementary figure 6: Histology for Figure 1, Supplementary figure 1 and Figure 3E-J.

(A) Histology for all fiber implants described in Figure **1A-J**. **(B)** Histology for all fiber implants described in Figure **1K-L** and **Supplementary figure 1**. **(C)** Histology for fiber implants located in the dHPC and the recording electrodes in the NAc for Figure **3E-J**.

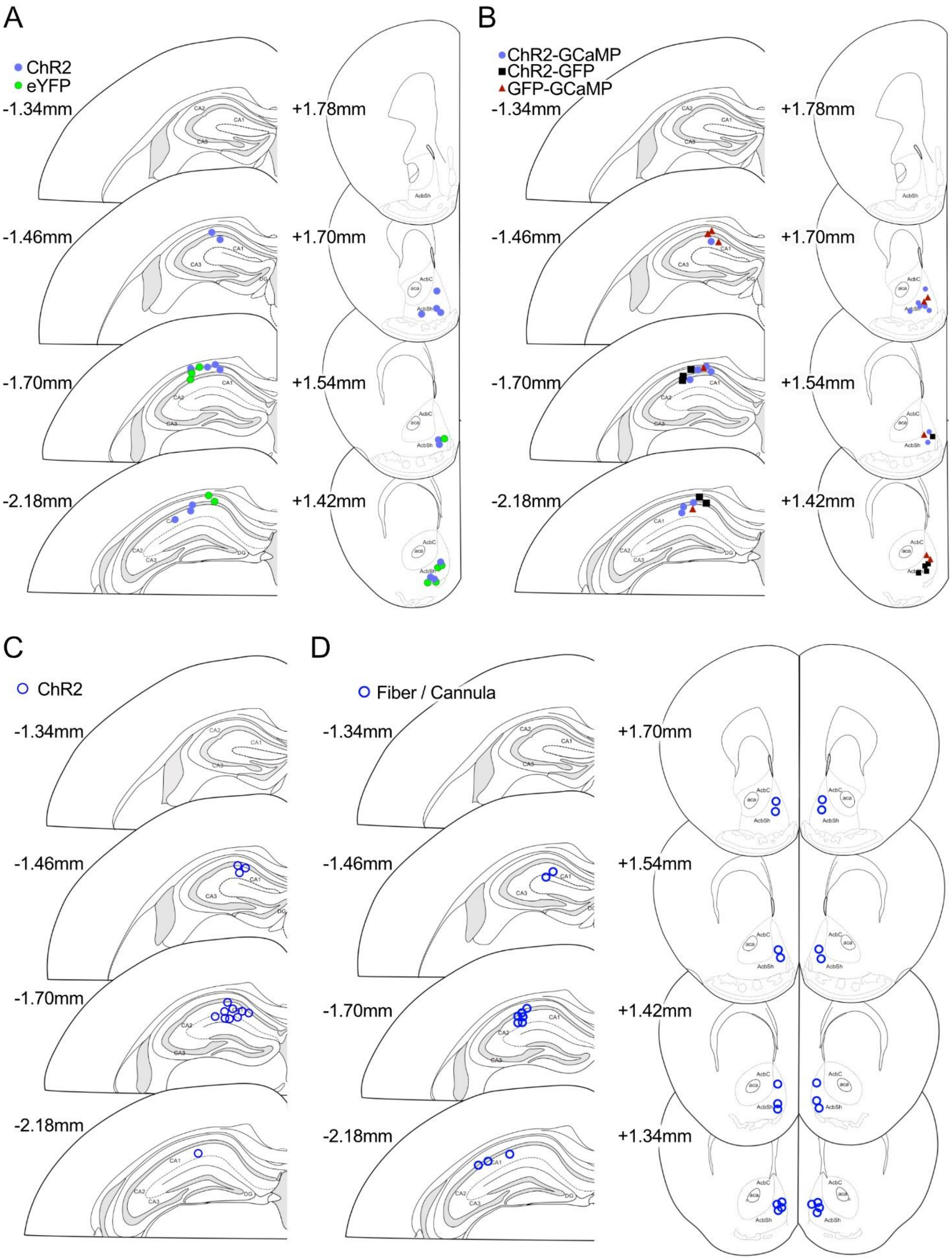
A
ChR2
eYFP
-1.34mm
-1.46mm
-1.70mm
-2.18mm
+1.78mm
+1.70mm
+1.54mm
+1.42mm
B
ChR2-GCaMP
ChR2-GFP
GFP-GCaMP
C
ChR2
D
Fiber / Cannula
+1.34mm
CA1
CA2
CA3
DG
AcbC
AcbSh
aca

Supplementary figure 7: Histology for Figure 3J-N, Supplementary figure 3 and Figure 4.

(A) Histology for all fiber implants in both the dHPC and NAcSh described in Figure **3J-N** and Supplementary figure **3A-D**. **(B)** Histology for all fiber implants in both dHPC and NAcSh described in Supplementary figure **3E-H**. **(C)** Histology for all fiber implants in Figure **4A-D**. **(D)** Histology for all fiber implants located in the dHPC and bilateral cannula in the NAcSh for Figure **4D-G**.

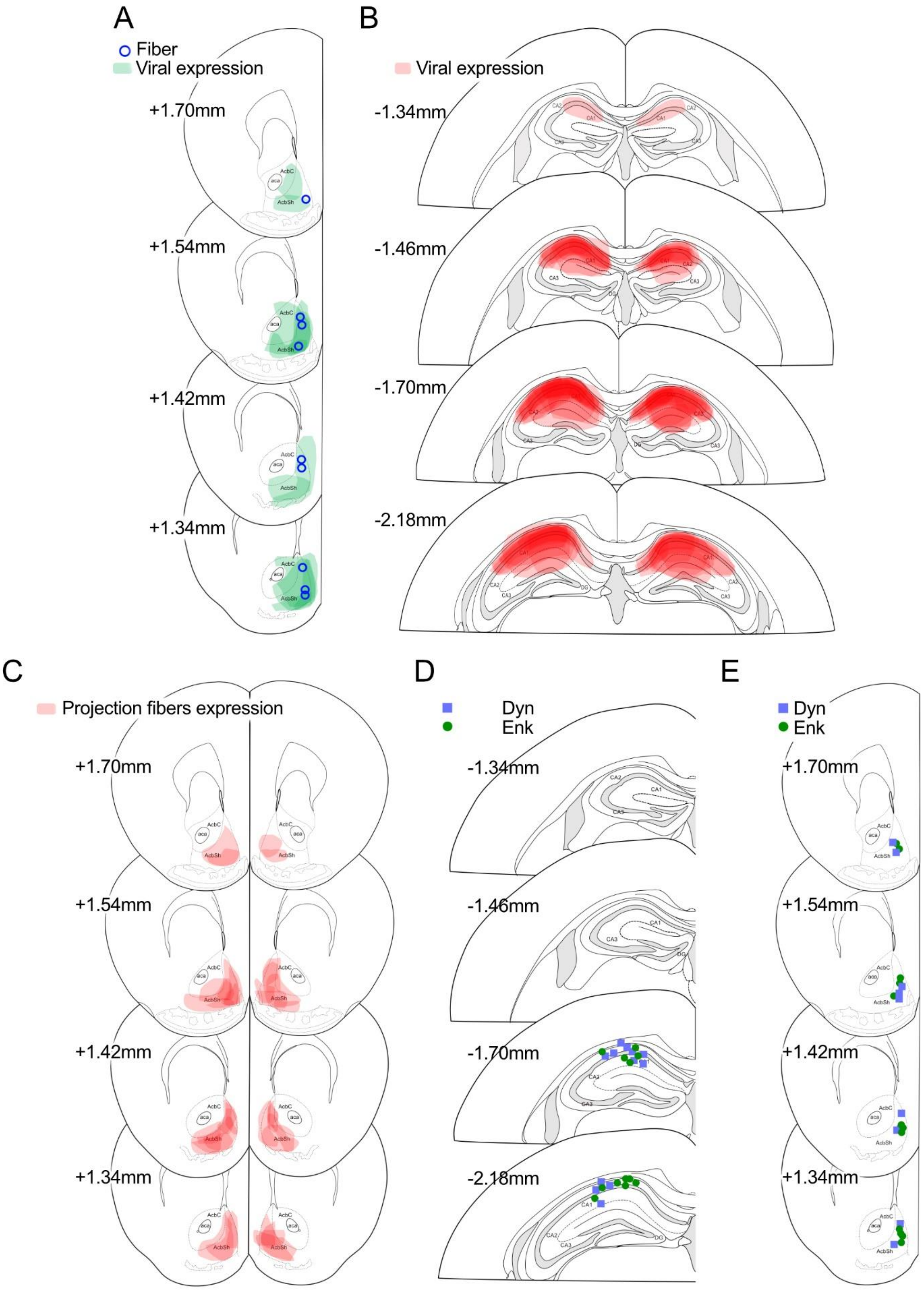
A
Fiber
Viral expression
+1.70mm
+1.54mm
+1.42mm
+1.34mm
B
Viral expression
-1.34mm
-1.46mm
-1.70mm
-2.18mm
C
Projection fibers expression
+1.70mm
+1.54mm
+1.42mm
+1.34mm
D
Dyn
Enk
-1.34mm
-1.46mm
-1.70mm
-2.18mm
E
Dyn
Enk
+1.70mm
+1.54mm
+1.42mm
+1.34mm

Supplementary figure 8: Histology for Supplementary figure 4, Figure 5 and Figure 6.

(A) Histology for viral expression and all fiber implants in the NAc described in Supplementary figure **4**. **(B)** Histology for viral expression in dHPC described in Figure **5**. **(C)** Histology for projection fibers expression in the NAc described in Figure 5. **(D)** Histology for all fiber implants in the dHPC described in Figure **6**. **(E)** Histology for all fiber implants in the NAcSh described in Figure **6**.

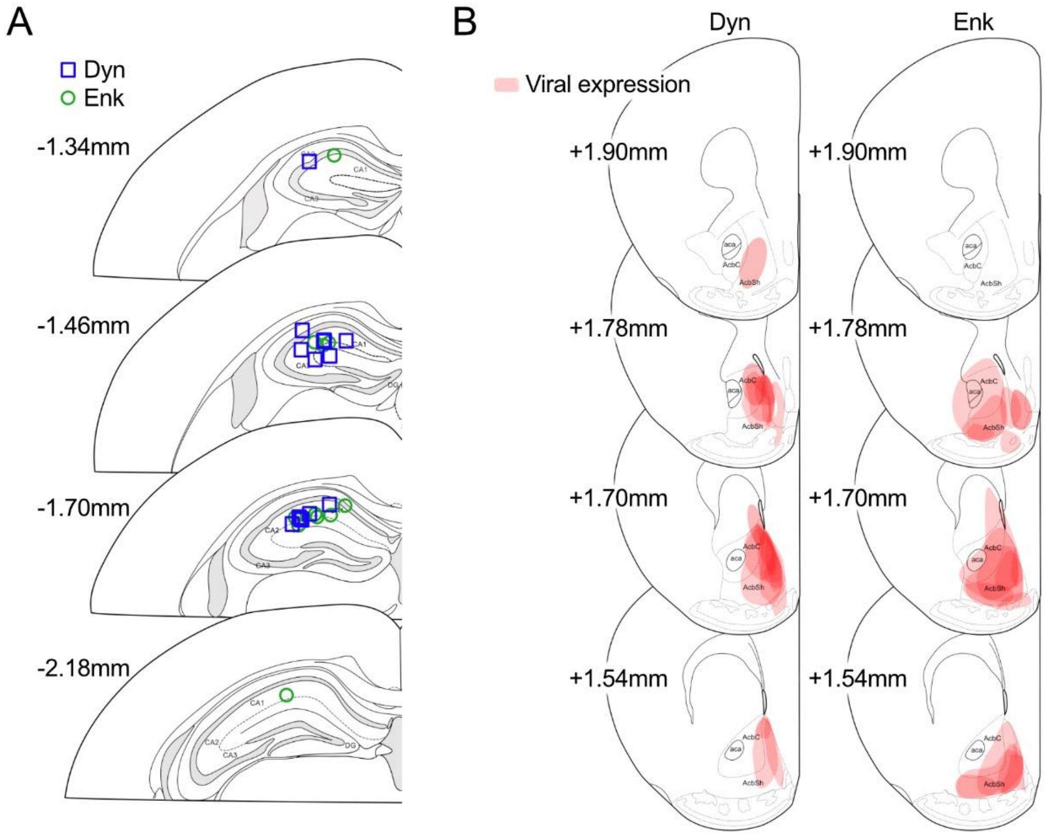

Supplementary figure 9: Histology for Figure 8 and Supplementary figure 5B-D.

(A) Histology for all fiber implants in the dHPC described in Figure **8** and Supplementary figure **5B-D**. **(B)** Histology for viral expression in the NAc in both Dyn- and Enk-cre mice described in Figure **8** and Supplementary figure **5B-D**.
