# Supplementary material for: Dorsal hippocampus to nucleus accumbens projections drive reinforcement via activation of accumbal dynorphin neurons
Source: Nat Commun. 2024 Jan 29;15:750. doi: 10.1038/s41467-024-44836-9 (PMC10825206; doi:10.1038/s41467-024-44836-9)
Supplement: Supplementary file 3 — Inventory of Supplementary Information [file 41467_2024_44836_MOESM3_ESM.docx]

Inventory of Supporting Information

1. Supplementary Figures in pdf format
   1. Name: 1-Suppl Figures_Dec2023
2. Statistics ouput from Prism for relevant main figures
   1. Name: 2-Main Fig Stats_Ibrahim_Nov23
3. Statistics ouput from Prism for relevant supplementary figures
   1. Name: 3-Suppl Fig Stats_Ibrahim_Nov23
4. Source file containing raw data for all the relevant main and supplementary figures
   1. Name: 4-Source file for dHPC-Nac Nat Comm 2023
