# Supplementary material for: Dorsal hippocampus to nucleus accumbens projections drive reinforcement via activation of accumbal dynorphin neurons
Source: Nat Commun. 2024 Jan 29;15:750. doi: 10.1038/s41467-024-44836-9 (PMC10825206; doi:10.1038/s41467-024-44836-9)
Supplement: Supplementary file 4 — Reporting Summary [file 41467_2024_44836_MOESM4_ESM.pdf]

nature portfolio

Corresponding author(s): J.A. Moron

Last updated by author(s): Dec 4, 2023

Reporting Summary

Nature Portfolio wishes to improve the reproducibility of the work that we publish. This form provides structure for consistency and transparency in reporting. For further information on Nature Portfolio policies, see our Editorial Policies and the Editorial Policy Checklist.

Statistics

For all statistical analyses, confirm that the following items are present in the figure legend, table legend, main text, or Methods section.

n/a	Confirmed	
☐	☒	The exact sample size (*n*) for each experimental group/condition, given as a discrete number and unit of measurement
☐	☒	A statement on whether measurements were taken from distinct samples or whether the same sample was measured repeatedly
☐	☒	The statistical test(s) used AND whether they are one- or two-sided<br>*Only common tests should be described solely by name; describe more complex techniques in the Methods section.*
☐	☒	A description of all covariates tested
☐	☒	A description of any assumptions or corrections, such as tests of normality and adjustment for multiple comparisons
☐	☒	A full description of the statistical parameters including central tendency (e.g. means) or other basic estimates (e.g. regression coefficient) AND variation (e.g. standard deviation) or associated estimates of uncertainty (e.g. confidence intervals)
☐	☒	For null hypothesis testing, the test statistic (e.g. *F*, *t*, *r*) with confidence intervals, effect sizes, degrees of freedom and *P* value noted<br>*Give P values as exact values whenever suitable.*
☒	☐	For Bayesian analysis, information on the choice of priors and Markov chain Monte Carlo settings
☒	☐	For hierarchical and complex designs, identification of the appropriate level for tests and full reporting of outcomes
☒	☐	Estimates of effect sizes (e.g. Cohen's *d*, Pearson's *r*), indicating how they were calculated

Our web collection on statistics for biologists contains articles on many of the points above.

Software and code

Policy information about availability of computer code

Data collection	Data was collected using the following softwares:<br>Med Associates Version 5 for self-stimulation experiments,<br>AnyMaze Version 7 for real time place testing,<br>Omniplex Plexon acquisition system (Version 1.18) for local field potential,<br>Synapse (Version 95; TDT) for fiber photometry, and<br>pClamp software Version 11.1 for whole-cell patch clamp recordings.<br>All experiments were performed in at least 2 cohorts of animals and treatment groups are repeated at least once in each cohort to avoid any unspecific day/condition effect.
Data analysis	Data were combined and statistics were ran through Prism - GraphPad software (Version 10).<br>NeuroExplorer (Version 5; Plexon Inc) were used to process and analyze local field potential data.<br>MatLab (Version 2019b) was used to process and analyze fiber photometry data. Custom codes and the usage of the code is available on https://github.com/khairunisa-ibrahim/Ibrahim-Massaly-2023

For manuscripts utilizing custom algorithms or software that are central to the research but not yet described in published literature, software must be made available to editors and reviewers. We strongly encourage code deposition in a community repository (e.g. GitHub). See the Nature Portfolio guidelines for submitting code & software for further information.

Data

Policy information about availability of data

All manuscripts must include a data availability statement. This statement should provide the following information, where applicable:

- Accession codes, unique identifiers, or web links for publicly available datasets
- A description of any restrictions on data availability
- For clinical datasets or third party data, please ensure that the statement adheres to our policy

Authors can confirm that all relevant data are included in the paper and its supplementary information files. Further information and requests for resources and reagents should be direct to will be fulfilled by the Lead Contact, Jose A. Moron (jmoron-concepcion@wustl.edu).

Research involving human participants, their data, or biological material

Policy information about studies with human participants or human data. See also policy information about sex, gender (identity/presentation), and sexual orientation and race, ethnicity and racism.

Reporting on sex and gender	N/A
Reporting on race, ethnicity, or other socially relevant groupings	N/A
Population characteristics	N/A
Recruitment	N/A
Ethics oversight	N/A

Note that full information on the approval of the study protocol must also be provided in the manuscript.

Field-specific reporting

Please select the one below that is the best fit for your research. If you are not sure, read the appropriate sections before making your selection.

☒ Life sciences ☐ Behavioural & social sciences ☐ Ecological, evolutionary & environmental sciences

For a reference copy of the document with all sections, see nature.com/documents/nr-reporting-summary-flat.pdf

Life sciences study design

All studies must disclose on these points even when the disclosure is negative.

Sample size	We performed a power analysis and incorporated approximately 20% attrition rate to select the number of animals used in each experiment (n = 12-14/group); this sample size account for attrition due to inadequate viral/cannula/fiber optic placement, and provide at least 80% power to detect effect sizes (Cohen's d), d > 1.2. at a significance level of p < 0.05.
Data exclusions	Animals were excluded on two criteria:<br>1. inadequate viral/ cannula/ fiber optic placement<br>2. lack of interaction with the active nose pokes during self-stimulation procedure. This lack of interaction did not allow the animals to exhibit reinforcing behavior as they never experienced self-stimulation.
Replication	All experiments were performed in at least 2 cohorts of animals and treatment groups are repeated at least once in each cohort to avoid any unspecific day/condition effect. All attempts of replication were successfully and included in the study.
Randomization	Treatments (i.e., choice of viral compounds with the dHPC and NAcSh) were randomly assigned to animals before testing.
Blinding	Experimenters were blind to treatment during data collection and statistical analysis, and results obtained by one experimenter were also analyzed by a second experimenter.

Reporting for specific materials, systems and methods

We require information from authors about some types of materials, experimental systems and methods used in many studies. Here, indicate whether each material, system or method listed is relevant to your study. If you are not sure if a list item applies to your research, read the appropriate section before selecting a response.

Materials & experimental systems

n/a	Involved in the study
☐	☒ Antibodies
☒	☐ Eukaryotic cell lines
☒	☐ Palaeontology and archaeology
☐	☒ Animals and other organisms
☒	☐ Clinical data
☒	☐ Dual use research of concern
☒	☐ Plants

Methods

n/a	Involved in the study
☒	☐ ChIP-seq
☒	☐ Flow cytometry
☒	☐ MRI-based neuroimaging

Antibodies

Antibodies used	Chicken anti-GFP (Abcam #ab13970, RRID: AB_300798)<br>Chicken anti-mCherry (Abcam #ab205402, RRID: AB_2722769)<br>Donkey anti-chicken Alexa Fluor 488 (Jackson ImmunoResearch #703-545-155, RRID: AB_2340375)<br>Donkey anti-chicken Cy3 (Jackson ImmunoResearch #730-165-155, RRID: AB_2340363)
Validation	All commercially available antibodies cited above were validated in previous peer-reviewed papers as mentioned in providers' website.<br>Please see web addresses below :<br>https://www.abcam.com/gfp-antibody-ab13970.html<br>https://www.abcam.com/products/primary-antibodies/mcherry-antibody-ab205402.html<br>https://www.jacksonimmuno.com/catalog/products/703-545-155<br>https://www.jacksonimmuno.com/catalog/products/703-165-155/Donkey-Chicken-IgG-HL-Cyanine-Cy3

Animals and other research organisms

Policy information about studies involving animals; ARRIVE guidelines recommended for reporting animal research, and Sex and Gender in Research

Laboratory animals	Adult WT, PEnk-IRES-Cre, and PDyn-IRES-Cre C57BL/6J male and female mice (20-30 g) were used for this study. All animals were 10 to 12 weeks at the beginning of the experiments. Four to five mice were housed together, given access to food pellets and water ad libitum, and maintained on a 12/12 h dark/light cycle (lights on at 7:00 AM). All animals were kept in a sound-attenuated, isolated holding facility in the lab 1 week prior to surgery, post-surgery, and throughout the duration of the behavioral assays to minimize stress. The housing conditionings were maintained at 20-26? degree Celsius with humidity levels at 30-70%.
Wild animals	No wild animals were used in the study.
Reporting on sex	We used a combination of male and female C57Bl6-J mice. All studies were conducted in both sexes. As no differences were observed in male vs female behavioral and fiber photometry outcomes, data was combined to strengthen power analysis.
Field-collected samples	No field-collected samples were used in the study.
Ethics oversight	All procedures were approved by the Washington University Institutional Animal Care and Use Committee (IACUC) in accordance with the National Institutes of Health Guidelines for the Care and Use of Laboratory Animals.

Note that full information on the approval of the study protocol must also be provided in the manuscript.

Plants

Seed stocks	N/A
Novel plant genotypes	N/A
Authentication	N/A
